# Empagliflozin Decreases Lactate Generation in an NHE-1 Dependent Fashion and Increases α-Ketoglutarate Synthesis From Palmitate in Type II Diabetic Mouse Hearts

**DOI:** 10.3389/fcvm.2020.592233

**Published:** 2020-12-04

**Authors:** Hong Zhang, Laween Uthman, Diane Bakker, Sahinda Sari, Sha Chen, Markus W. Hollmann, Ruben Coronel, Nina C. Weber, Sander M. Houten, Michel van Weeghel, Coert J. Zuurbier

**Affiliations:** ^1^Laboratory of Experimental Intensive Care and Anesthesiology, Department of Anesthesiology, Amsterdam Cardiovascular Sciences, Amsterdam Universitair Medische Centra, University of Amsterdam, Amsterdam, Netherlands; ^2^Department of Anesthesiology, The Second Affiliated Hospital of Xi'an JiaoTong University, Xi'an, China; ^3^Clinical and Experimental Cardiology, Amsterdam Cardiovascular Sciences, Amsterdam Universitair Medische Centra, University of Amsterdam, Amsterdam, Netherlands; ^4^Department of Genetics and Genomic Sciences, Icahn Institute for Data Science and Genomic Technology, Icahn School of Medicine at Mount Sinai, New York, NY, United States; ^5^Laboratory Genetic Metabolic Diseases, Amsterdam Gastroenterology and Metabolism, Amsterdam Cardiovascular Sciences, Amsterdam Universitair Medische Centra, University of Amsterdam, Amsterdam, Netherlands; ^6^Core Facility Metabolomics, Amsterdam Universitair Medische Centra, University of Amsterdam, Amsterdam, Netherlands

**Keywords:** SGLT2, glycolysis, glucose oxidation, fatty acid oxidation, NHE, diastolic function, oxygen consumption, isolated heart

## Abstract

**Aims/hypothesis:** Changes in cardiac metabolism and ion homeostasis precede and drive cardiac remodeling and heart failure development. We previously demonstrated that sodium/glucose cotransporter 2 inhibitors (SGLT2i's) have direct cardiac effects on ion homeostasis, possibly through inhibition of the cardiac sodium/hydrogen exchanger (NHE-1). Here, we hypothesize that Empagliflozin (EMPA) also possesses direct and acute cardiac effects on glucose and fatty acid metabolism of isolated type II diabetes mellitus (*db/db*) mouse hearts. In addition, we explore whether direct effects on glucose metabolism are nullified in the presence of an NHE-1 inhibitor.

**Methods:** Langendorff-perfused type II diabetic db/db mouse hearts were examined in three different series: **1**: ^13^C glucose perfusions (*n* = 32); **2**: ^13^C palmitate perfusions (*n* = 13); and **3**: ^13^C glucose + 10 μM Cariporide (specific NHE-1 inhibitor) perfusions (*n* = 17). Within each series, EMPA treated hearts (1 μM EMPA) were compared with vehicle-perfused hearts (0.02% DMSO). Afterwards, hearts were snap frozen and lysed for stable isotope analysis and metabolomics using LC-MS techniques. Hearts from series 1 were also analyzed for phosphorylation status of AKT, STAT3, AMPK, ERK, and eNOS (*n* = 8 per group).

**Results:** Cardiac mechanical performance, oxygen consumption and protein phosphorylation were not altered by 35 min EMPA treatment. EMPA was without an overall acute and direct effect on glucose or fatty acid metabolism. However, EMPA did specifically decrease cardiac lactate labeling in the ^13^C glucose perfusions (^13^C labeling of lactate: 58 ± 2% vs. 50 ± 3%, for vehicle and EMPA, respectively; *P* = 0.02), without changes in other glucose metabolic pathways. In contrast, EMPA increased cardiac labeling in α-ketoglutarate derived from ^13^C palmitate perfusions (^13^C labeling of α-KG: 79 ± 1% vs. 86 ± 1% for vehicle and EMPA, respectively; *P* = 0.01). Inhibition of the NHE by Cariporide abolished EMPA effects on lactate labeling from ^13^C glucose.

**Conclusions:** The present study shows for the first time that the SGLT2 inhibitor Empagliflozin has acute specific metabolic effects in isolated diabetic hearts, i.e., decreased lactate generation from labeled glucose and increased α-ketoglutarate synthesis from labeled palmitate. The decreased lactate generation by EMPA seems to be mediated through NHE-1 inhibition.

## Introduction

Sodium glucose cotransport 2 inhibitors (SGLT2i's) significantly reduce cardiovascular risk in diabetic patients. However, the underlying molecular mechanisms of the beneficial cardiovascular effects of SGLT2i's remain incompletely understood. Changes in cardiac metabolism and ion homeostasis precede and drive cardiac remodeling and heart failure development upon metabolic or hemodynamic overload (1–4). We previously demonstrated that SGLT2's exert direct and acute effects on ion homeostasis of the heart, by reducing intracellular Na^+^ and Ca^2+^ possibly through inhibition of the cardiac Na^+^/H^+^ exchanger (NHE) (5, 6). It is currently unknown, whether SGLT2i's also exert direct and acute effects on substrate metabolism of the isolated diabetic heart. Although other studies (7, 8) have examined SGLT2i's effects on cardiac metabolism, these studies involved chronic *in vivo* SGLT2i administration, without proper control for the systemic glucose effects of SGLT2i's and/or studying metabolism of the isolated heart without SGLT2i actually being present in the perfusate. Thus, the primary goal of the present study is to characterize whether SGLT2i can directly affect cardiac metabolism of the isolated diabetic heart. Secondly, we (5, 6) and others (9–11) found evidence that SGLT2i's may, at least partly, mediate their direct cellular effects through NHE-1 inhibition. A similar NHE-1 inhibition by empagliflozin was recently observed for human failing heart cardiomyocytes (12). SGLT2i inhibition of NHE results in intracellular ion changes of Na^+^ and Ca^2+^ (5, 6) that may precipitate in changes in mitochondrial function (13) and can reprogram cardiac metabolism (14). Therefore, as a secondary goal, we examined whether possible SGLT2i effects on cardiac glucose metabolism are mediated through NHE-1 inhibition, by also studying SGLT2i effects in the presence of an NHE-1 inhibitor. Finally, effects of SGLT2i on survival proteins reported to facilitate cardioprotection against ischemic insult (15), and occasionally reported to be affected by SGLT2i's in isolated cells or in the *in vivo* condition (e.g., STAT3, AMPK, Akt, eNOS) (16), were also examined for the isolated heart. Therefore, in the present study we hypothesized that the SGLT2i Empagliflozin (EMPA) affects glucose and fatty acid metabolism in the isolated diabetic mouse heart, with possible involvement of cardiac NHE and/or of survival proteins.

## Methods

Animal handling was in accordance with the Institutional Animal Care and Use Committee of Amsterdam UMC, location AMC, and was performed in accordance with guidelines from Directive 2010/63/EU of the European Parliament on the protection of animals used for scientific purposes.

## Animals

Male db/db mice (11–14 weeks old; BKS.Cg-Dock7^m^+/+Lepr^db^/J; Charles River) were housed with a maximum of 7 mice per cage for at least 1 week in our institute, with a 12 h day/night cycle, and food (Teklad global 16% protein rodent diet (Envigo, Indianapolis, IN, US) and drinking water *ad libitum*. Within each group 1–3, animals within each treatment group were matched to time-of-day.

## Heart Perfusions

Mice were injected i.p., with heparin (15 mU) and anesthetized with 125 mg/kg S(+)-ketamine and 0.2 mg/kg dexmedetomidine. Anesthetic depth was tested with the pedal withdrawal reflex. Mice were intratracheally ventilated with 50% O_2_ and 50% N_2_, the chest was opened, and the heart aorta was in-chest cannulated for immediately perfusion of hearts.

Hearts were Langendorff-perfused at a constant flow (initial perfusion pressure of 65 mmHg) with Krebs-Henseleit buffer containing (in mmol/l): 118 NaCl, 4.7 KCl, 2.5 CaCl_2_, 1.2 MgSO_4_, 25 NaHCO_3_, 1.2 KH_2_PO_4_, 0.5 EDTA, 5.5 glucose, 1 lactate, 0.1 pyruvate, 0.5 glutamine, 1% albumin-0.4 mM palmitate, 0.05 L-carnitine, 100 mU/L insulin and 5 nmol/l epinephrine, and gassed with 95% O_2_/5% CO_2_. The perfusate was in-line filtered by a 0.45 μm filter. An apex cannula was pierced through the left ventricular wall for release of Thebesian flow. For all hearts end-diastolic pressure (EDP) was set between 2 and 5 mm Hg by adjusting balloon volume, and perfusion pressure set at 65 mm Hg during the first 20 min stabilization period. Hearts were discarded when heart rate <280 beats/min, left ventricular developed pressure <70 mm Hg or when displaying an irregular heartbeat. All hearts were continuously submerged in 37°C perfusate.

LV developed pressure (DLVP) was calculated from peak systolic pressure minus end-diastolic pressure. The Rate-Pressure-Product (RPP, index of mechanical performance) was the product of the developed pressure and heart rate. At 25 min, the pulmonary artery was sampled to determine venous oxygen partial pressure; at 35 min, the arterial inflow (after removal of the heart) was sampled to determine arterial oxygen partial pressure. Samples were analyzed immediately using a blood gas analyzer (RapidPoint400; Siemens). Oxygen consumption was derived from (arterial-venous oxygen pressure) times the measured flow (ml/min/G dry weight).

EMPA (MedChem Express, Monmouth Junction, NJ, USA) or DMSO, dissolved in Milli-Q-water, were delivered with a minipump (Minipulse, Harvard) at 1% of total perfusion flow through a side-arm connected to an in-line custom-made mixing chamber. Drugs administration started at baseline (*t* = 0 min), resulting that hearts were subjected to the drugs at approximately *t* = 3 min of the 35 min perfusion protocol (traveling time from side-arm to heart). For the cariporide series, the Krebs-Henseleit buffer contained 10 μmol/l cariporide (Aventis Pharma, Frankfurt, Germany). [U-^13^C_6_] glucose (initial molar percent enrichment (MPE): 99%; Cambridge Isotope Laboratories, Andover, USA) was dissolved at 5.5 mmol/l in KHB at the experimental day, whereas [U-^13^C_16_] palmitate (MPE:98%; Cambridge Isotope Laboratories, Andover, USA) was bound to albumin, dialyzed and filtered before the experimental day.

## Experimental Groups

Three groups of mice were studied: **1** ^13^C glucose perfusions (*n* = 32); **2**: ^13^C palmitate perfusions (*n* = 13); and **3**: ^13^C glucose + 10 μM Cariporide (specific NHE-1 inhibitor) perfusions (*n* = 17).

After 20 min stabilization, DMSO (0.02% final concentration) or DMSO+EMPA (1 μM final concentration) was administered to the hearts through a side arm with mixing chamber just above the heart. The hearts were then perfused for the next 35 min. At 35 min, hearts were quickly frozen in liquid nitrogen and freeze-dried overnight. Parts of freeze-dried hearts were prepared for and analyzed by Liquid Chromatography-Mass Spectrometry (LC-MS, see below). Within each group, hearts were randomized to time-of-day for both treatments. Experimenters were blinded to group assignment during data analysis.

## LC-MS

Half of the hearts from group 1 (16 hearts total), and all hearts from group 2 (13 hearts total) and group 3 (17 hearts total) were subjected to LC-MS analysis. The first half of group 1 hearts could not be subjected to the final LC-MS, because the LC-MS technique was not fully developed at that moment. Metabolomics was performed as previously described, with minor adjustments (17). Samples were freeze dried, crunched and ~2 mg weighted in a 2 mL tube. A 75 μL mixture of internal standard adenosine-^15^N_5_-monophosphate (AMP-15N; 100 μM) was added to each sample. Subsequently, 425 μL water, 500 μL methanol and 1 mL chloroform were added to the same 2 mL tube before thorough mixing and centrifugation for 10 min at 14.000 rpm. The top layer, containing the polar phase, was transferred to a new 1.5 mL tube and dried using a vacuum concentrator at 60°C. Dried samples were reconstituted in 100 μL methanol/water (6/4; v/v). Metabolites were analyzed using a Waters Acquity ultra-high-performance liquid chromatography system coupled to a Bruker Impact II™ Ultra-High Resolution Qq-Time-Of-Flight mass spectrometer. Samples were kept at 12°C during analysis and 5 μL of each sample was injected. Chromatographic separation was achieved using a Merck Millipore SeQuant ZIC-cHILIC column (PEEK 100 × 2.1 mm, 3 μm particle size). Column temperature was held at 30°C. Mobile phase consisted of (A) 1:9 acetonitrile:water and (B) 9:1 acetonitrile:water, both containing 5 mM ammonium acetate. Using a flow rate of 0.25 mL/min, the LC gradient consisted of: 100% B for 0–2 min, ramp to 0% B at 28 min, 0% B for 28–30 min, ramp to 100% B at 31 min, 100% B for 31-35 min. MS data were acquired using negative and positive ionization in full scan mode over the range of m/z 50–1,200. Data were analyzed using Bruker TASQ software version 2.1.22.3. Isotope ratios and natural background corrections were calculated using IsoCorrectoR (18). Metabolite identification has been based on a combination of accurate mass, (relative) retention times and fragmentation spectra, compared to the analysis of a library of standards. Data are presented as total label % or total metabolite content. Total label % entails the label incorporation in a metabolite corrected for natural abundance and tracer impurities using the R package IsocorrectoR (18), calculated as the sum of the areas of M1 to Mx isotopologues relative to the sum of areas of M0 to Mx. Total metabolite content equals the raw areas of M0 to Mx isotopologues of each metabolite summed up and normalized to the raw area of the internal standard AMP-15N and dry weight heart sample.

## Western Blotting Survival Proteins

5–10 mg freeze-dried heart tissue, from heart of group 1, was homogenized in cold buffer, containing 0.02 M HEPES, 0.25 M sucrose, 1 mM dithiothreitol (DTT) and phosphatase and protease inhibitor cocktail on ice. The samples were sonicated for 2 × 4 s on ice and lysed with 0.5% Triton for 10 min at room temperature. Afterwards, the samples were centrifuged at 10,000 g for 1 min at 4 °C. The supernatant was stored at −80°C for determining signaling proteins.

Protein concentration in supernatant was determined with Lowry assay. Western blot was conducted as described previously. Briefly, equal amounts of protein were run through 4–12% sodium dodecyl sulfate polyacrylamide (SDS) gel (Biorad) and transferred to polyvinylidene fluoride (PVDF) membrane. The membranes were incubated in Odyssey blocking buffer at room temperature for 1 h and probed with antibodies for phospho-Akt (Ser473) (1:1000; CST #9271), Akt (1:1000; CST #9272), Phospho-eNOS (Ser1177) (1:1000; CST #9571), eNOS (1:1000; CST #9572), phospho-p44/42 MAPK (Erk1/2) (1:1000; CST #9101), p44/42 MAPK (Erk1/2) (1:1000; CST #9102), phosphor-STAT3 (Thr705) (1:1000; CST #9131), STAT3 (1:1000; CST #9139), phospho-AMPKα (Thr172) (1:1000; CST #2535), AMPKα (1:1000; CST #2603), phospho-Acetyl-CoA Carboxylase (Ser79) (1:1000; CST #11818), Acetyl-CoA Carboxylase (C83B10) (1:1000; CST #3676), PDH (1:1000; CST #2784s), phospho-PDH E1a (Ser293) (1:2000; Sigma AP1062) and GAPDH (1:5000; abcam #9485). Signal of bands was obtained and quantified by Odyssey system (Li-cor).

### Statistics

Data are reported as mean ± SE. Normality distribution was tested by Shapiro–Wilk test. If data were normally distributed, Student's *t*-test was performed for comparison. Otherwise Mann–Whitney *U* tests were applied. For metabolic pathways that were represented by more than one intermediate metabolite (i.e., glycolysis, pentose phosphate pathway and TCA cycle) multiple testing was performed with Bonferroni correction. Statistical analysis was conducted using IBM SPSS statistics version 24 (International Business Machines Corp., Armond, NY, USA). Figures were made in GraphPad Prism 8.0 (GraphPad Software, Inc., La Jolla, CA, USA). A value of *P* < 0.05 was considered statistically different.

## Results

### EMPA Reduces Cardiac Lactate Generation and Increases α-Ketoglutarate Synthesis Without Affecting Cardiac Performance, Oxygen Consumption or Phosphorylation Status of Survival Proteins

#### Group 1

**Figure 1:**
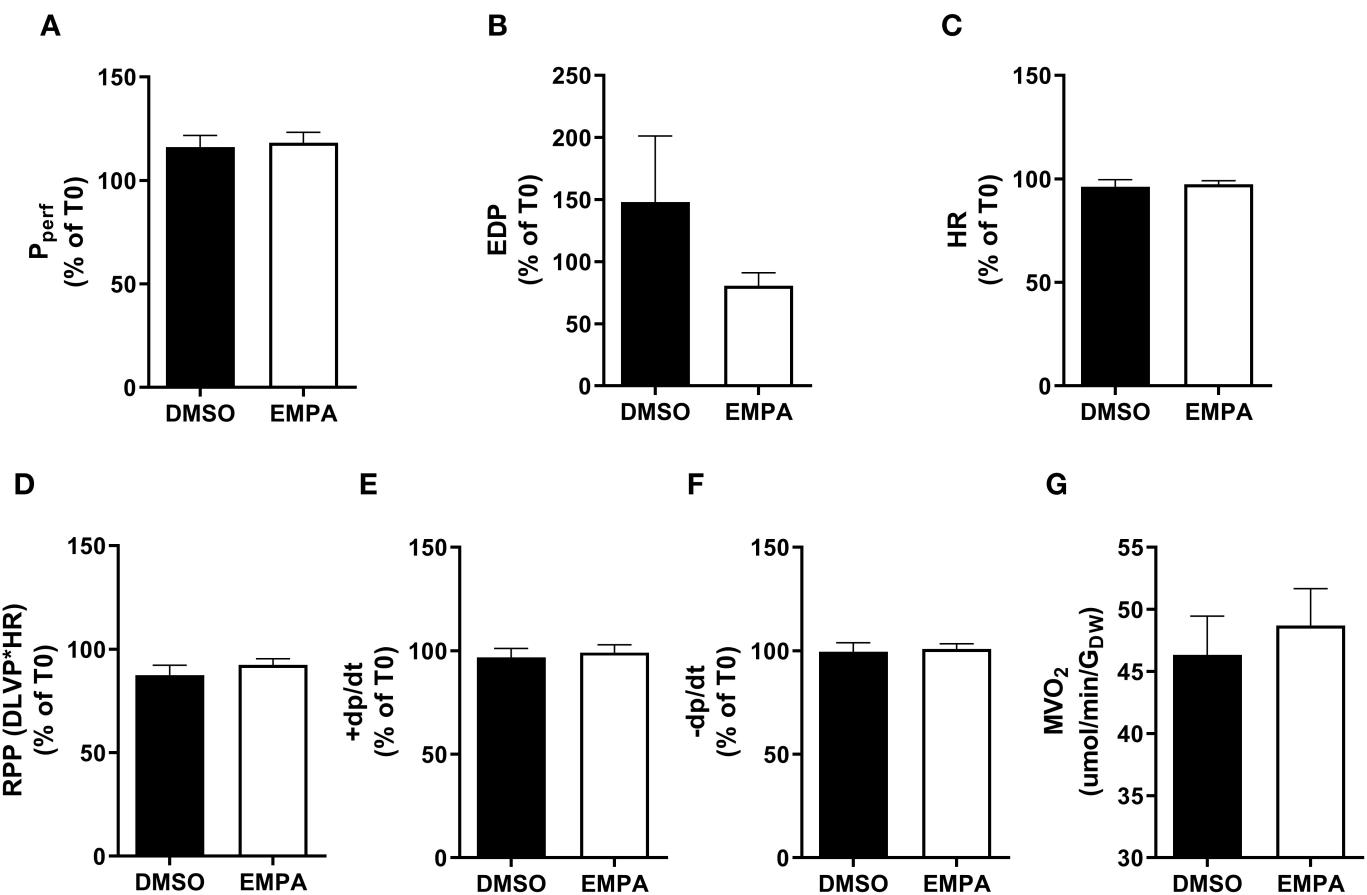
EMPA's effects on heart function and oxygen consumption with ^13^C-glucose in perfusate in group 1. For all cardiac function parameters, the change at T = 35 min relative to the value at T = 0 min is depicted. **(A)** Perfusion pressure (P_perf_), **(B)** end diastolic pressure (EDP), **(C)** heart rate (HR), **(D)** rate pressure product (RPP); DLVP, developed left ventricular pressure, **(E)** maximum contraction rate of left ventricle (+dp/dt), and **(F)** maximum relaxation rate of left ventricle (-dp/dt), **(G)** oxygen consumption rate (MVO_2_) determined at 25 min perfusion. *N* = 16 for DMSO and *n* = 16 for EMPA. All values represent mean ± SEM.

Baseline (before EMPA administration) cardiac performance of isolated db/db hearts between DMSO and EMPA group was not significantly different (Supplementary Table 1A). Perfusion with EMPA did not change coronary vascular resistance, end-diastolic left ventricular pressure, heart rate, rate-pressure-product or activation and relaxation rate of left ventricle of the diabetic heart (Figures 1A–F). EMPA was also without effect on cardiac oxygen consumption (Figure 1G).

**Figure 2:**
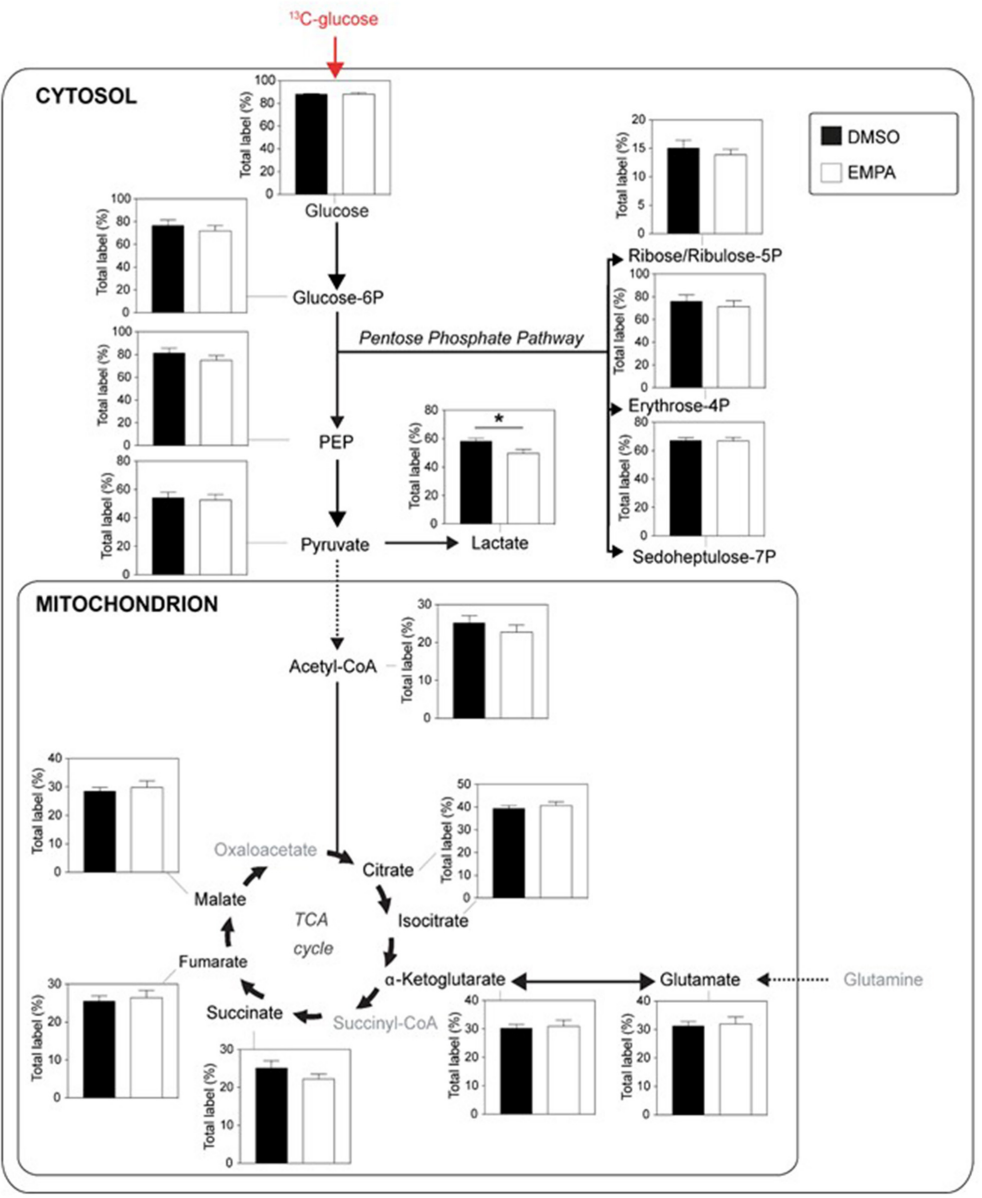
EMPA's direct effects on glucose metabolism of db/db hearts. EMPA's effects on ^13^C-labeling in metabolites of glycolysis, lactate generation, pentose phosphate pathway and TCA cycle when hearts were perfused with 5.5 mM ^13^C glucose; total label % = % metabolite labeled with ^13^C; ppp = pentose phosphate pathway; DMSO (*n* = 8), EMPA (*n* = 8); *P < 0.05.

Next, we examined the incorporation of ^13^C from ^13^C glucose into glucose metabolic pathways of the heart, i.e., glucose uptake (% labeling glucose), glycolysis (% labeling G6P, PEP and pyruvate), lactate production (% labeling lactate), pentose phosphate pathway (PPP; % labeling in ribose/ribulose-5P, erythrose-4P and sedoheptulose-7P), pyruvate dehydrogenase activity (% labeling in acetylCoA) and glucose oxidation (% labeling in TCA cycle intermediates) (Figure 2). EMPA was without effect on glucose uptake, glycolysis and PPP. EMPA administration did, however, significantly lowered ^13^C glucose incorporation into lactate. Analysis of total lactate in the effluent of the hearts revealed no effect of Empa (0.98±0.02 mM vs. 0.96±0.01 mM, for DMSO vs. EMPA, respectively; *p* = NS). Perfusion with ^13^C glucose resulted in ~25% labeling of TCA intermediates, indicating glucose contribution to TCA cycle in these db/db hearts. However, EMPA was without effect on ^13^C glucose labeling of acetylCoA and TCA intermediates, suggesting that Empa was without a direct and acute overall effect on glucose oxidation in the diabetic heart. Phosphorylation status of pyruvate dehydrogenase, a crucial control point regulation glucose going into acetylCoA, was unaffected by EMPA treatment (Supplementary Figure 1). Also, EMPA did not change the phosphorylation status of Akt, eNOS, Erk, STAT3, AMPK and ACC (Supplementary Figure 2).

#### Group 2

We then studied EMPA effects on cardiac function and metabolism using ^13^C palmitate perfusions. As in group 1, baseline (before EMPA administration) cardiac performance of isolated db/db hearts between DMSO and EMPA group was not significantly different (Supplementary Table 1B). Perfusion with EMPA did not change cardiac performance or oxygen consumption (Supplementary Figure 3).

**Figure 3:**
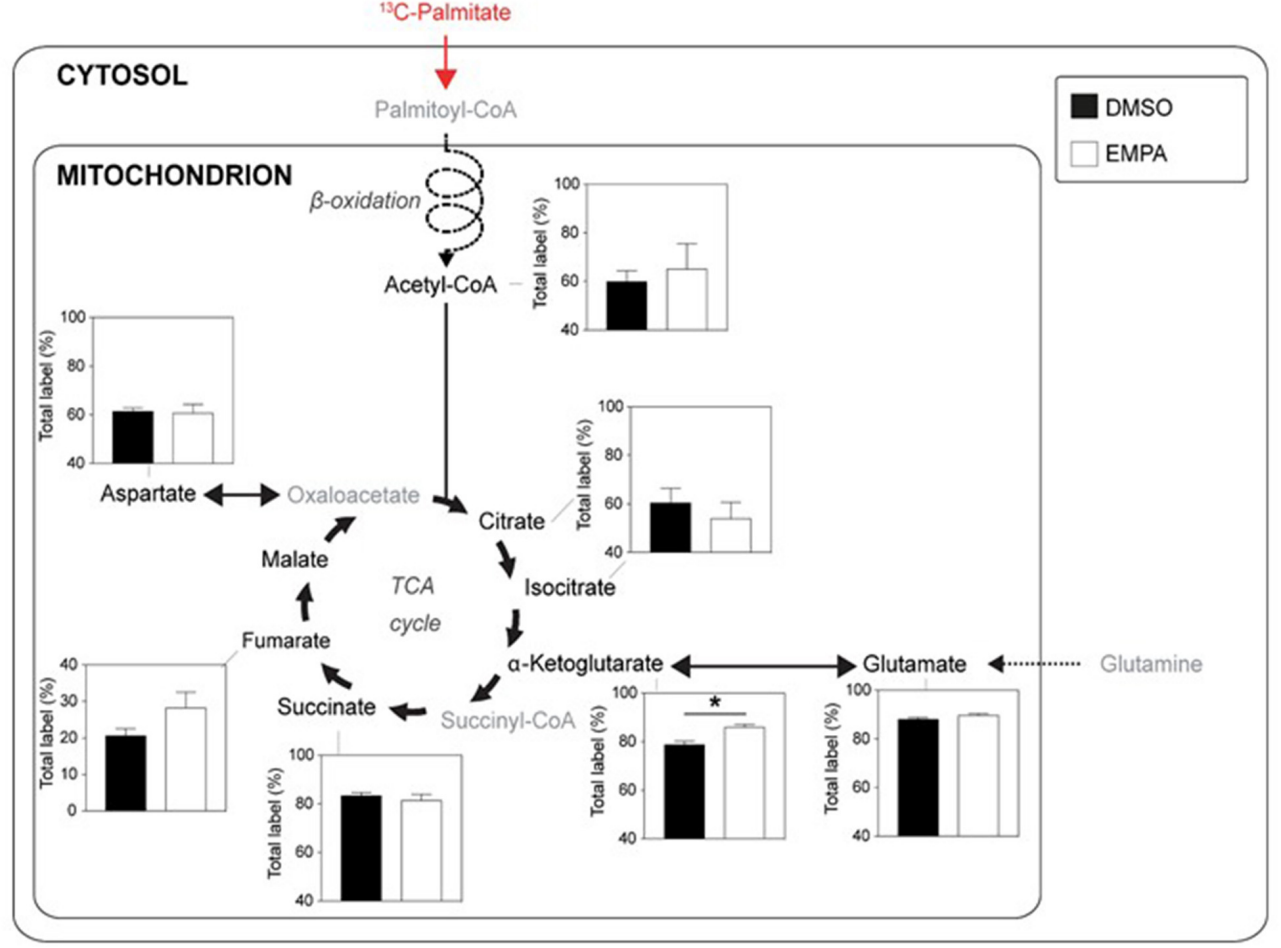
EMPA's direct effects on palmitate metabolism of db/db hearts. ^13^C-labeling in metabolites of the TCA cycle when hearts were perfused with 0.4 mM ^13^C palmitate; DMSO (*n* = 5), EMPA (*n* = 8), % = % metabolite labeled with ^13^C; *P < 0.05.

Perfusion with ^13^C palmitate resulted in ~60% labeling of TCA intermediates, indicating that fatty acids contributed 2–3 times more than glucose to TCA cycle activity (Figure 3). EMPA was without effect on ^13^C palmitate labeling for most intermediates of the TCA cycle, indicating no overall acute effect of EMPA on cardiac fatty acid metabolism. However, ^13^C incorporation from ^13^C palmitate into α-ketoglutarate (Figure 3) was significantly increased following EMPA treatment.

Collectively, these data show that acute EMPA specifically reduces cardiac lactate generation from glucose and increases cardiac α-ketoglutarate synthesis from palmitate, without alterations in overall glucose or fatty acid metabolism, cardiac function or oxygen consumption. We showed previously that EMPA changes ion homeostasis through NHE inhibition (5, 6). Therefore, we next investigated whether the effects of EMPA on glucose metabolism were also dependent on NHE activity.

### NHE Inhibition by Cariporide Prevents Decreased Lactate Generation by EMPA and Attenuates EMPA Lowering of Metabolic Intermediates

#### Group 3

**Figure 4:**
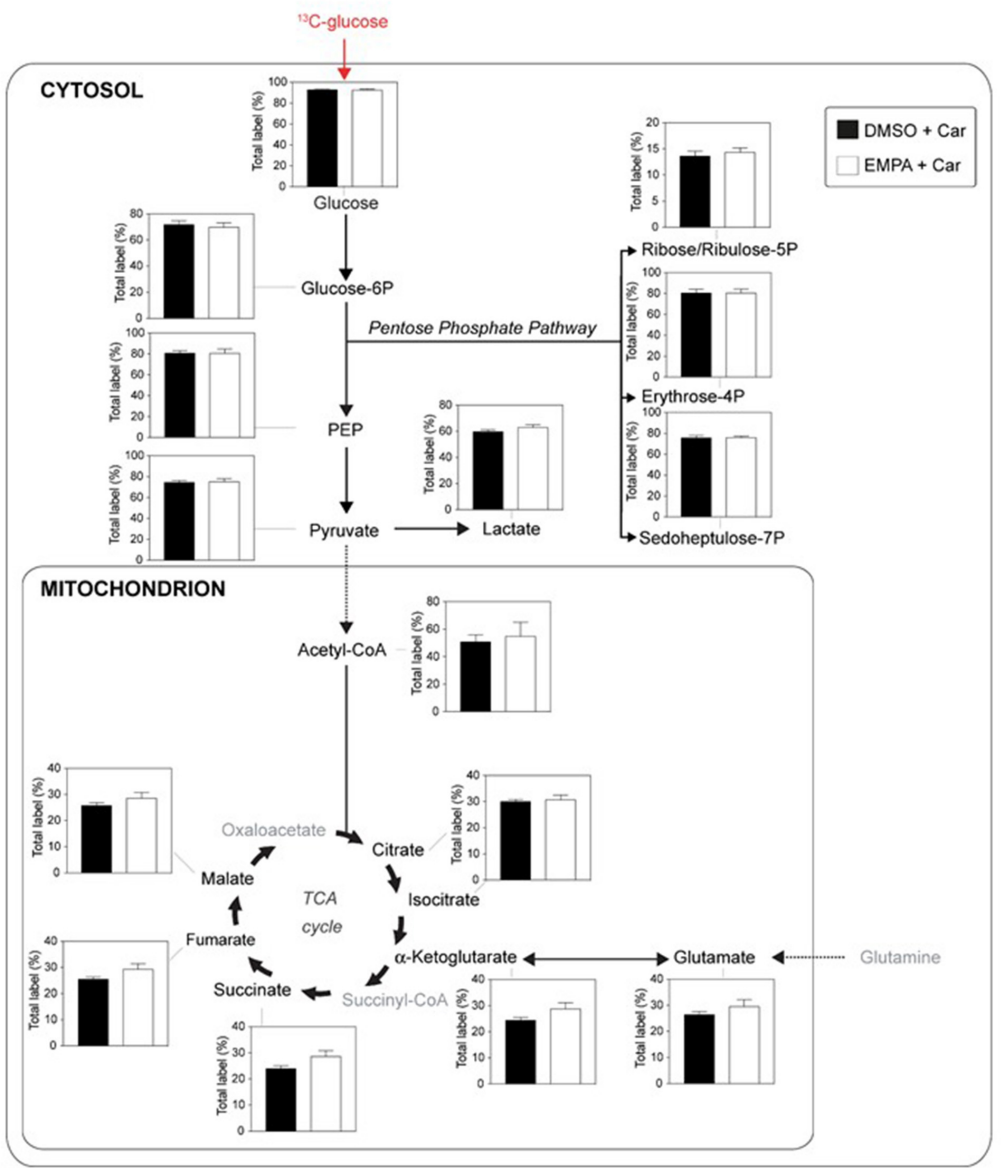
NHE-1 inhibition (cariporide) prevents EMPA's effects on glucose ^13^C labeling of lactate in db/db hearts. EMPA's effects on ^13^C-labeling in metabolites of glycolysis, pentose phosphate pathway and TCA cycle when hearts were perfused with 5.5. mM ^13^C glucose and 10 μM cariporide; DMSO (*n* = 9), EMPA (*n* = 8), % = % metabolite labeled with ^13^C; ppp = pentose phosphate pathway.

We examined EMPA effects on cardiac function and metabolism using ^13^C glucose perfusions in the presence of the NHE-1 inhibitor Cariporide (10 μM). Baseline (before EMPA administration) cardiac performance of isolated db/db hearts between DMSO and EMPA group was not significantly different (Supplementary Table 1C). Perfusion with EMPA was largely without effect on cardiac performance and oxygen consumption (Supplementary Figure 4), except for an EMPA-induced increase in contractile activation rate (Supplementary Figure 4E) and relaxation rate (Supplementary Figure 4F). Figure 4 displays ^13^C glucose labeling in glucose metabolic pathways for hearts treated with Cariporide, showing that NHE-1 inhibition by Cariporide prevented the EMPA-induced decrease in ^13^C glucose labeling in lactate.

**Figure 5:**
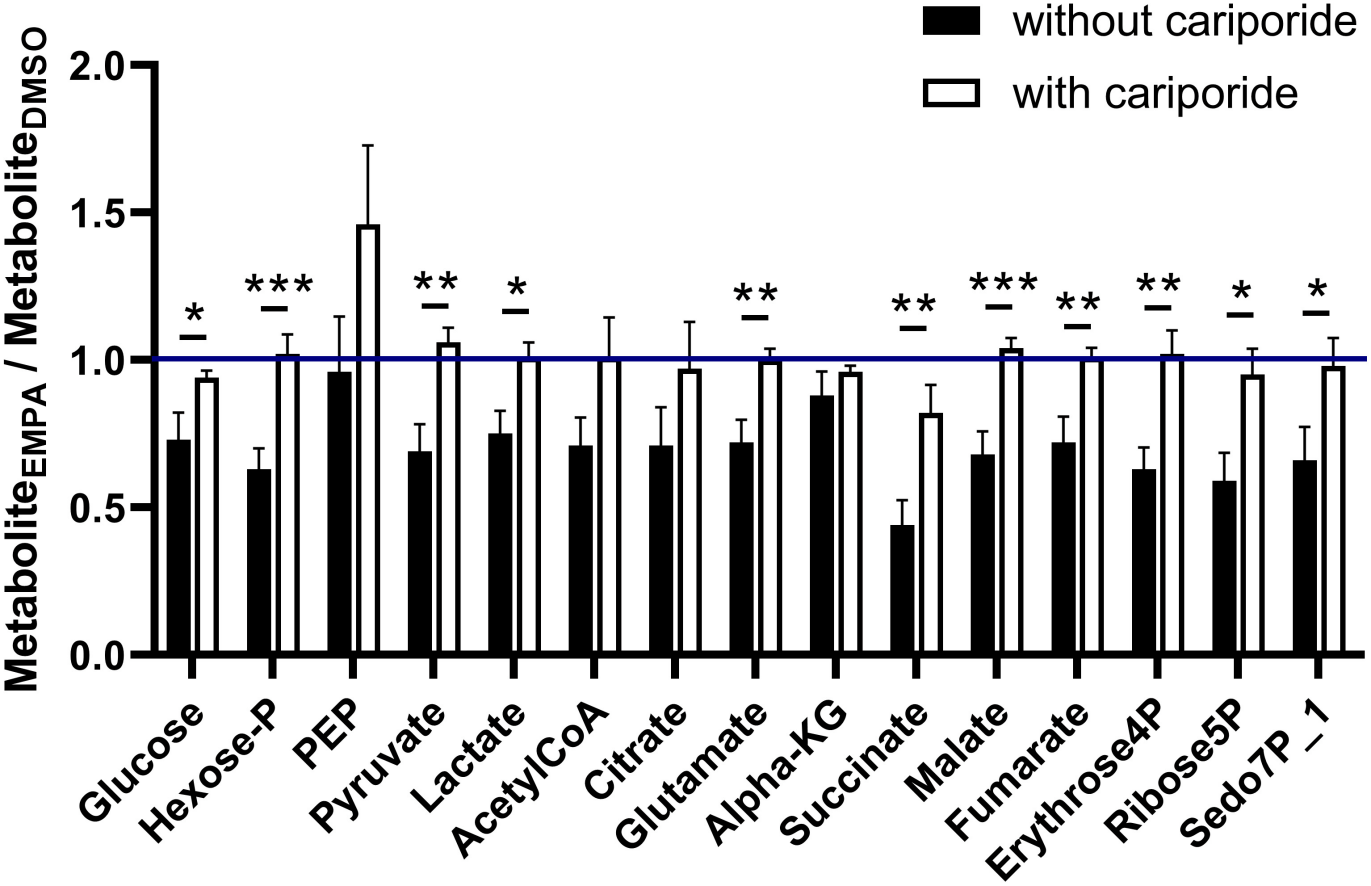
NHE-1 inhibition (cariporide) attenuates Empa's lowering effect on metabolic intermediate levels of glucose metabolism. Cariporide's effects on EMPA-induced relative changes in total (unlabeled plus labeled) metabolite content (AU) in the ^13^C glucose perfusions. DMSO (*n* = 8), EMPA (*n* = 8), DMSO + Cariporide (*n* = 9), EMPA + Cariporide (*n* = 8); *P < 0.05, **P < 0.01, ***P < 0.001.

Finally, we examined whether NHE-1 inhibition affected EMPA effects on total (unlabeled plus labeled) metabolic intermediates in the ^13^C glucose perfusions without and with Cariporide (Figure 5). Without NHE inhibition, EMPA administration lowered glucose intermediary metabolites by about 20–30%, whereas NHE-1 inhibition significantly attenuated most of EMPA's reducing effects on metabolite concentrations. These data suggest that EMPA's effects on lactate generation and cardiac metabolite levels are in general mediated through NHE-1 inhibition.

## Discussion

We report here that the SGLT2i Empagliflozin has a direct effect on specific, not overall, metabolic pathways of the isolated mouse diabetic heart. These changes in cardiac metabolism cannot be ascribed to whole-body diuretic, metabolic (increased ketones) or kidney-dependent processes of EMPA, because we used an isolated heart preparation. Thus, EMPA reduces cardiac lactate generation from external glucose and increases α-ketoglutarate production from external fatty acid. The cardiac lactate effects of EMPA were absent in the presence of an NHE-1 inhibitor, suggesting that EMPA mediates this metabolic effect through modulation of NHE-1, probably through the lowering of cardiac intracellular sodium and calcium. Indeed, our previous work showed that SGLT2i's directly reduce cardiac intracellular Na^+^ (5, 6). It was recently shown that increases in intracellular Na^+^ cause overall fatty acid metabolism to shift to glucose metabolism (14). Although the observed effects in the present study thus also showed some partly lowering of glucose metabolism and some partly increasing of fatty acid metabolism with acute EMPA treatment, no overall shift in metabolism between glucose and fatty acid was observed as in (14). One likely explanation is that EMPA only lowers intracellular Na^+^ by 1–2 mM (5, 6), whereas the imposed increase in intracellular Na^+^ that induced the overall shifting of glucose and fatty acid metabolism was with 15 mM much larger (14). Further studies will be necessary to directly monitor intracellular Na^+^ within the intact heart during acute and more chronic treatment of EMPA to elucidate to what extent EMPA effects on cardiac metabolism are mediated through changes in intracellular Na^+^.

Although other studies have examined whether SGLT2i's may change cardiac metabolism, interpretation of these studies is cumbersome. In one study (7), EMPA's effects were examined in severely fasted animals, creating conditions that are optimal for raising systemic ketone levels with SGLT2i's (7), but which do not mimic the normal, non-fasting conditions, that are present in life. In another study, a 4 week EMPA treatment was compared with a non-glucose treatment in db/db mice, resulting in large chronic differences in blood glucose levels which confound the results, and then comparing the isolated hearts in the absence of EMPA (8). In the latter study of isolated db/db heart studies (8), no significant changes in metabolic pathways were observed following chronic EMPA treatment. Thus, our study is the first to report acute and direct beneficial cardiac metabolic effects induced by EMPA.

## EMPA, Lactate and α-Ketoglutarate (αKG) Generation

A recent study has reported decreased lactate release in epicardial fat cells from coronary artery disease patients following 6-h treatment with the SGLT2i dapagliflozin, which was associated with alterations in gene expression (19). Changes in gene expressions are unlikely in the current study due to the short 35 min treatment period. Lactate generation can also be a sign of an energy-deprived heart (lactate as biochemical ischemia marker), and therefore lowering of lactate may result from an improved energetic status of the heart by EMPA. However, this also seems unlikely because we were unable to find changes in the phosphorylation status of AMPK, and cardiac function and oxygen consumption were not changed by EMPA. This excludes ischemia as a causative factor. To the best of our knowledge, this is the first study reporting that SGLT2i increases αKG synthesis from exogenous fatty acids within the heart. Intriguingly, increased kidney αKG generation with EMPA was recently observed in kidneys of non-diabetic mice (20). Although we have not examined the mechanisms underlying this increased production, an activation of the mitochondrial isocitrate dehydrogenase enzyme, the enzyme producing α-KG, by mitochondrial Ca^2+^ might explain the observed finding. EMPA has been reported to increase mitochondrial Ca^2+^ (5), and this enzyme is under the control of mitochondrial Ca^2+^ (21). Further research will be necessary to test this hypothesis. Increases in αKG may be beneficial in cardiac pathology, because αKG was reported to elicit anti-inflammatory actions (22).

## Cardiac Metabolism and Heart Failure

Metabolic changes precede and drive cardiac remodeling and heart failure development (23, 24). Our results are in line with these findings, in that the observed metabolic changes by acute EMPA were not associated with cardiac functional changes. Previous work showed that acute EMPA was also without direct cardiac effects in healthy mouse hearts (6, 25). If metabolic alterations drive cardiac pathology, it will also suggest that clinical diagnosis of cardiovascular compromise may be improved by adding metabolic evaluation next to the now common functional evaluation of cardiac pathology. Early detection of increased risk of cardiovascular disease may thus improve prognosis and aid in timely treatment. Cardiac hypertrophy has been associated with enhanced glycolysis whereby the glycolytic intermediates provide the building blocks to facilitate cell growth. Indeed, G6P accumulation has recently been shown to be an important activator of mTOR, the master molecular switch of protein synthesis (26). In addition, the shift away from fatty acid metabolism is associated with the development of heart failure (26). The observed acute metabolic effects of EMPA reported in the present study (decreased G6P, metabolism shifting from lactate production to partly fatty acid metabolism) demonstrate that EMPA may be able to prevent these detrimental cardiac metabolic changes that drive heart failure development. Further studies are necessary to test whether these initial cardiac metabolic changes induced by SGLT2i's in the diabetic heart, also are present in other types of diseased hearts, and can indeed contribute to SGLT2i's beneficial heart failure modulating effects observed in several clinical trials.

In conclusion, the results demonstrate that EMPA has direct cardiac metabolic effects in the diabetic heart. EMPA, at least for glucose metabolism mediated by cardiac NHE-1, decreases lactate generation from glucose and increases α-ketoglutarate from palmitate, suggestive of a partly shift from glucose to fatty acid metabolism in the diabetic heart.

## Data Availability Statement

The raw data supporting the conclusions of this article will be made available by the authors, without undue reservation.

## Ethics Statement

All animal experiments were approved by the Animal Ethics Committee of the Academic Medical Center, Amsterdam, The Netherlands and performed in accordance with guidelines from Directive 2010/63/EU of the European Parliament on the protection of animals used for scientific purposes.

## Author Contributions

HZ and CZ designed the study. HZ, LU, and DB performed experiments. HZ, LU, SS, SC, SH, MW, and CZ provided materials, performed measurements, and analyzed data. HZ and CZ wrote the manuscript. LU, RC, MH, NW, SH, and MW revised critically the manuscript. All authors approved final version of manuscript submitted.

## Conflict of Interest

The authors declare that the research was conducted in the absence of any commercial or financial relationships that could be construed as a potential conflict of interest.
