# Supplementary material for: Empagliflozin Decreases Lactate Generation in an NHE-1 Dependent Fashion and Increases α-Ketoglutarate Synthesis From Palmitate in Type II Diabetic Mouse Hearts
Source: Front Cardiovasc Med. 2020 Dec 4;7:592233. doi: 10.3389/fcvm.2020.592233 (PMC7746656; doi:10.3389/fcvm.2020.592233)
Supplement: Supplementary file 1 [file Table_1.DOCX]

**Electronic Supplementary Materials (ESM)**

**Figure 1**

EMPA’s effects on pyruvate dehydrogenase (PDH) activity in hearts of series 1. **(A/B)** Representative immunoblots and analysis of phospho-PDH and total PDH. N = 8 per group. All values represent mean ± SEM.

**Figure 2**

EMPA’s effects on cardioprotective signaling pathways after 35 min perfusion from hearts of series 1. **(A)** Representative immunoblots and analysis of phospho-Akt and total Akt, **(B)** representative immunoblots and analysis of phospho-eNOS and total eNOS, **(C)** representative immunoblots and analysis of phospho-Erk and total Erk, **(D)** representative immunoblots and analysis of phospho-STAT3 and total STAT3, **(E)** representative immunoblots and analysis of phospho-AMPK and total AMPK, **(F)** representative immunoblots and analysis of phospho-ACC and total ACC. N = 8 per group. All values represent mean ± SEM.

**Figure 3**

EMPA’s effects on heart function and cardiac oxygen consumption with ^13^C-palmitate in perfusate in series 2. For all cardiac function parameters, the change at T = 35 min relative to the value at T = 0 min is depicted. **(A)** Perfusion pressure (P_perf_), **(B)** end diastolic pressure (EDP), **(C)** heart rate (HR), **(D)** rate pressure product (RPP); DLVP, developed left ventricular pressure, **(E)** maximum contraction rate of left ventricle (+dp/dt) and **(F)** maximum relaxation rate of left ventricle (-dp/dt). **(G)** oxygen consumption rate (MVO_2_) determined at 25 min perfusion. For cardiac function, n = 6 for DMSO and n = 8 for EMPA; for oxygen consumption, n = 5 for DMSO and n = 8 for EMPA. All values represent mean ± SEM.

**Figure 4**

EMPA’s effects on heart function after 35 min perfusion under cariporide treatment in series 3. For all parameters, the change at T = 35 min relative to the value at T = 0 min is depicted. **(A)** Perfusion pressure (P_perf_), **(B)** end diastolic pressure (EDP), **(C)** heart rate (HR), **(D)** rate pressure product (RPP); DLVP, developed left ventricular pressure, **(E)** maximum contraction rate of left ventricle (+dp/dt) and **(F)** maximum relaxation rate of left ventricle (-dp/dt). **(G)** oxygen consumption rate (MVO_2_) determined at 25 min perfusion. For cardiac function, n = 9 for DMSO and n = 8 for EMPA; for oxygen consumption, n = 8 for DMSO and n = 7 for EMPA. Cari, cariporide; All values represent mean ± SEM. **P* < 0.05.

**Figure 1**

**
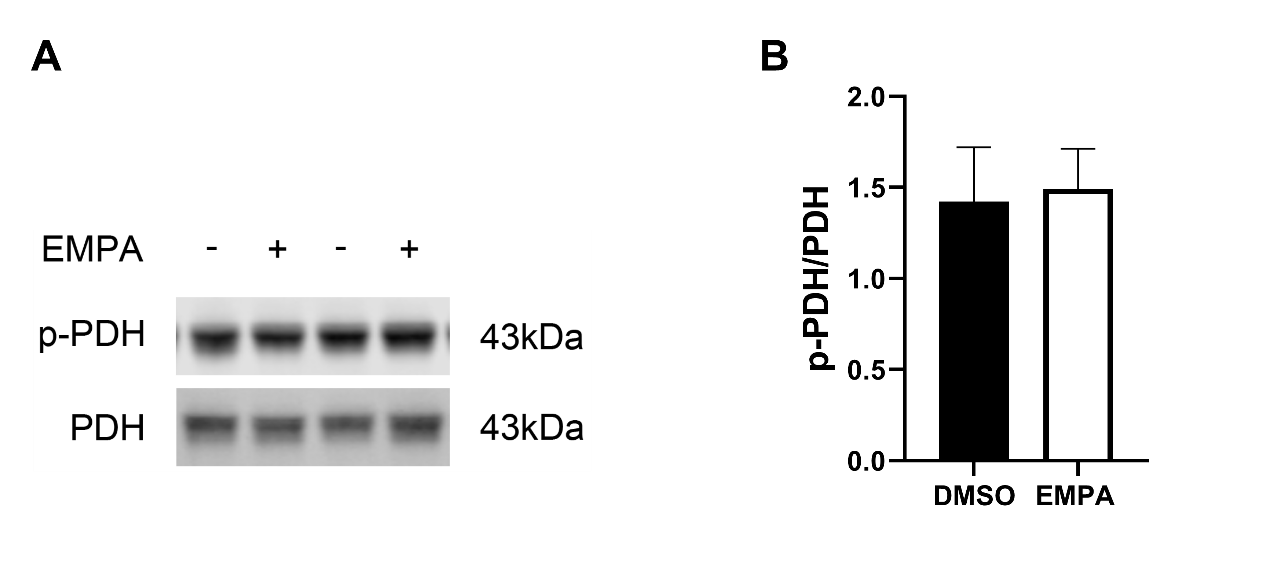
**

**Figure 2**


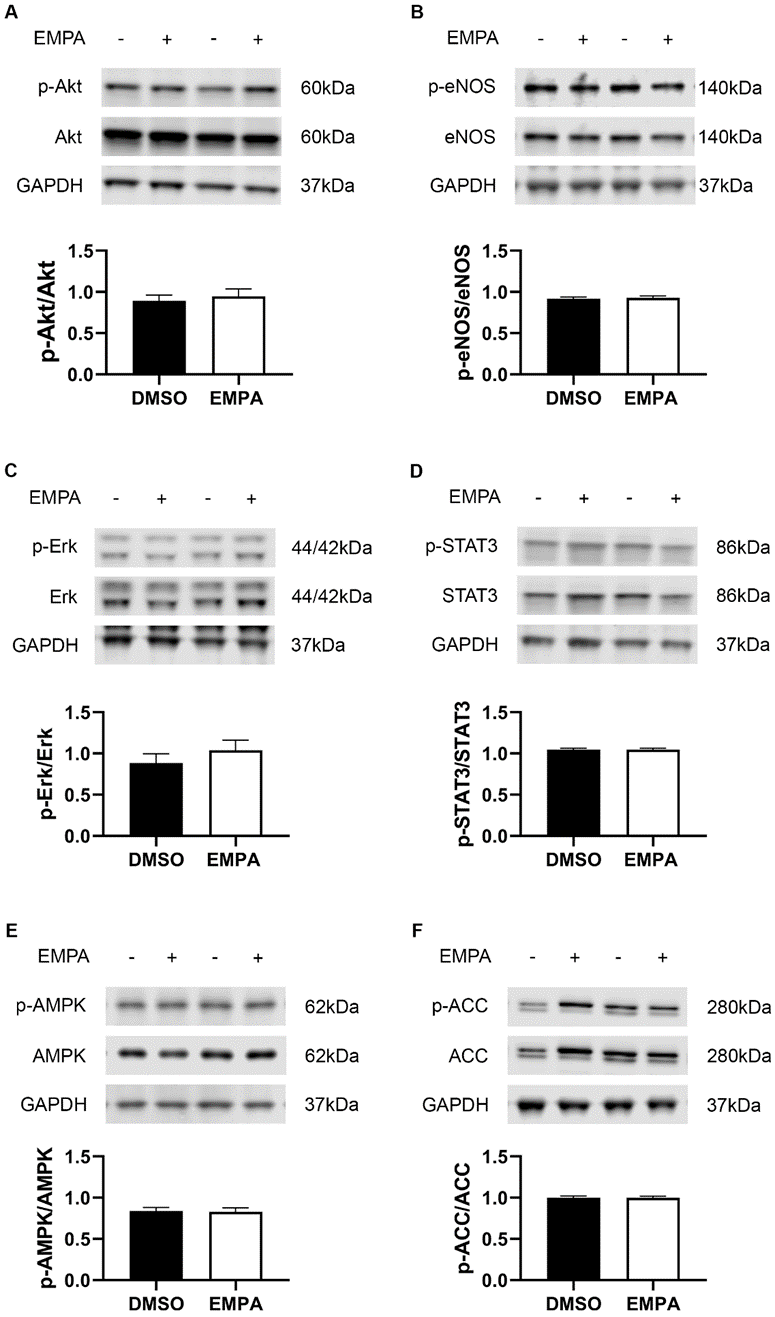


**Figure 3**


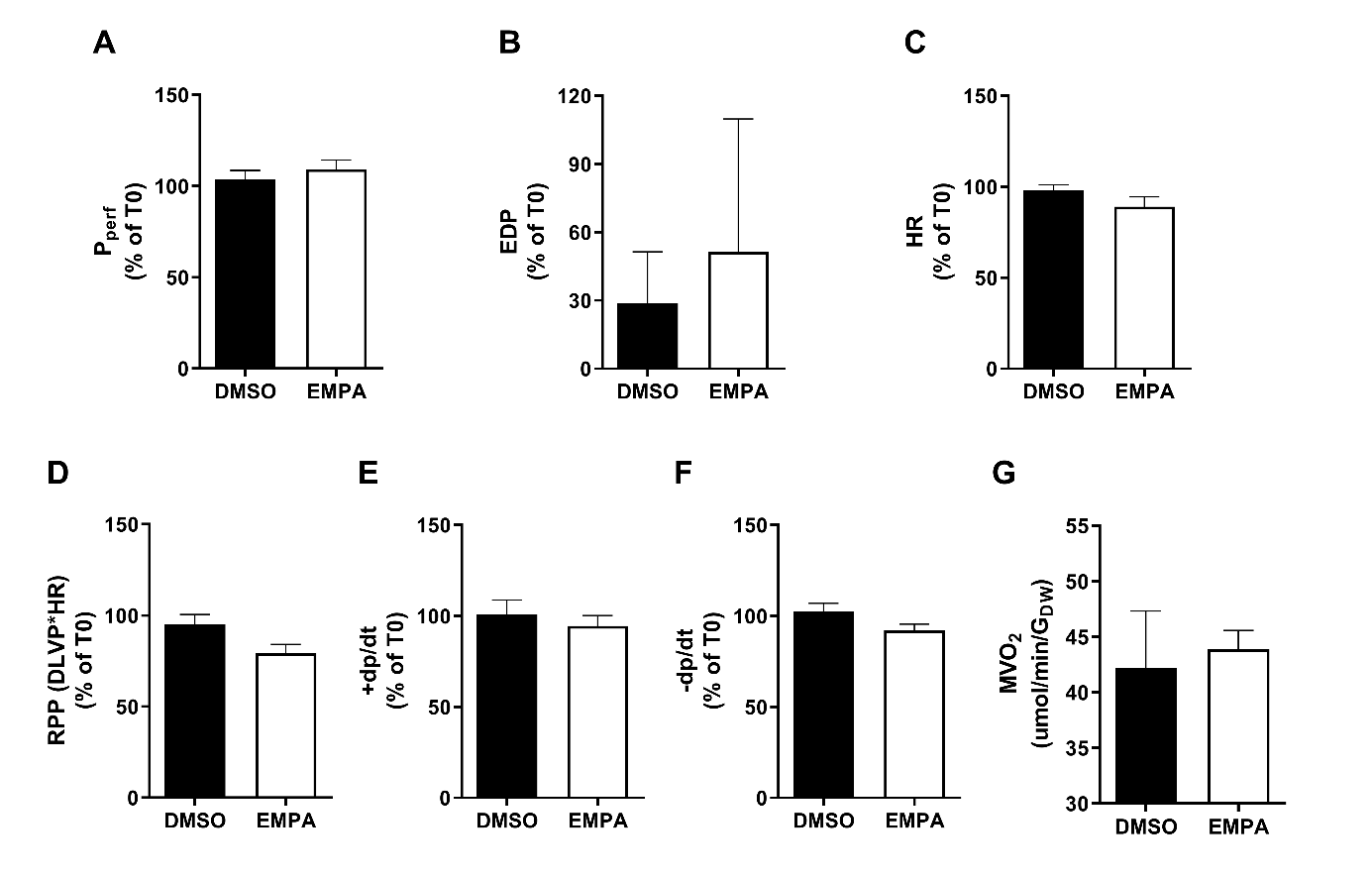


**Figure 4**


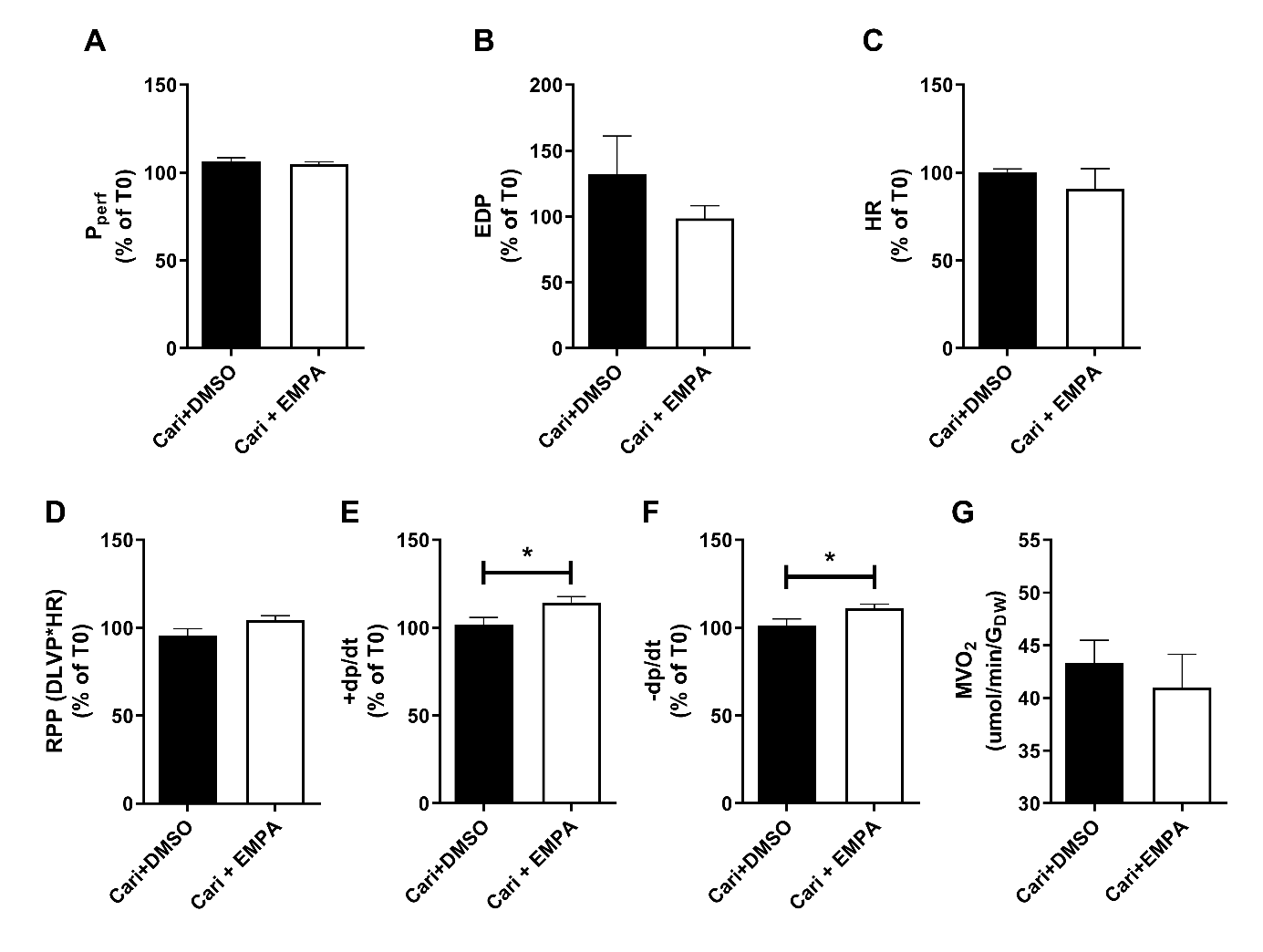


**Table 1**

| **Table 1A (^13^C glucose)** | | |
| --- | --- | --- |
|  | DMSO (n=16) | EMPA (n=16) |
| Body weight (g) | 47.7±0.5 | 47.3±0.6 |
| Heart dry weight (HDW; mg) | 30±1 | 29±1 |
| P_perf_ (mmHg) | 65±1 | 65±1 |
| Flow (ml/min/G_DW_) | 75±5 | 80±5 |
| EDP (mmHg) | 2.6±0.2 | 3.6±0.4 |
| DLVP (mmHg) | 139±6 | 140±5 |
| HR (beats/min) | 360±10 | 357±5 |
| RPP (DLVP*HR) | 49793±1986 | 49601±1279 |
| +dp/dt (mmHg/s) | 5631±247 | 5584±185 |
| -dp/dt (mmHg/s) | 4790±207 | 4663±161 |

| **Table 1B (^13^C palmitate)** | | |
| --- | --- | --- |
|  | DMSO (n=6) | EMPA (n=8) |
| Body weight (g) | 47.4±0.9 | 46.2±1.1 |
| Heart dry weight (HDW; mg) | 29±1 | 27±1 |
| P_perf_ (mmHg) | 63±1 | 65±1 |
| Flow (ml/min/G_DW_) | 74±7 | 74±2 |
| EDP (mmHg) | 2.8±0.5 | 3.5±0.5 |
| DLVP (mmHg) | 141±8 | 142±4 |
| HR (beats/min) | 339±18 | 362±11 |
| RPP (DLVP*HR) | 47965±4017 | 51158±1540 |
| +dp/dt (mmHg/s) | 5891±485 | 5819±255 |
| -dp/dt (mmHg/s) | 4977±310 | 5126±94 |

| **Table 1C (^13^C glucose + cariporide)** | | |
| --- | --- | --- |
|  | DMSO (n=9) | EMPA (n=8) |
| Body weight (g) | 45.8±1.1 | 45.2±0.9 |
| Heart dry weight (HDW; mg) | 28±0 | 28±1 |
| P_perf_ (mmHg) | 61±1 | 62±1 |
| Flow (ml/min/G_DW_) | 64±2 | 65±5 |
| EDP (mmHg) | 3.3±0.3 | 3.2±0.5 |
| DLVP (mmHg) | 140±6 | 134±4 |
| HR (beats/min) | 342±9 | 355±9 |
| RPP (DLVP*HR) | 47711±1563 | 47540±1792 |
| +dp/dt (mmHg/s) | 5620±323 | 5291±170 |
| -dp/dt (mmHg/s) | 4664±176 | 4630±123 |

Baseline characteristics at T = 0 in ^13^C glucose, ^13^C palmitate and ^13^C glucose + cariporide series, respectively. P_perf_, perfusion pressure; EDP, end diastolic pressure; DLVP, developed left ventricular pressure; HR, heart rate; RPP, rate pressure product; +dp/dt, maximum contraction rate of left ventricle; and -dp/dt, maximum relaxation rate of left ventricle. All values represent mean ± SEM. Statistical analysis was by *t* test or Mann-Whitney *U* test.
